# Predictors of sickness absence and intention to leave the profession among NHS staff in England during the COVID-19 pandemic: a prospective cohort study

**DOI:** 10.1136/bmjopen-2024-097483

**Published:** 2025-06-04

**Authors:** Lauren J Scott, Danielle Lamb, Chris Penfold, Maria Theresa Redaniel, Nora Trompeter, Paul Moran, Rupa Bhundia, Neil Greenberg, Rosalind Raine, Simon Wessely, Ira Madan, Peter Aitken, Anne Marie Rafferty, Sarah Dorrington, Richard Morriss, Dominic Murphy, Sharon A M Stevelink

**Affiliations:** 1 National Institute for Health and Care Research Applied Research Collaboration West (NIHR ARC West), University Hospitals Bristol and Weston NHS Foundation Trust, Bristol, UK; 2 Population Health Sciences, University of Bristol Medical School, Bristol, UK; 3 Department of Applied Health Research, University College London, London, UK; 4 Institute of Psychiatry, Psychology & Neuroscience, King’s College London, London, UK; 5 Guy's and St Thomas’ Hospitals NHS Trust, London, UK; 6 Sussex Partnership NHS Foundation Trust, Sussex, UK; 7 Florence Nightingale Faculty of Nursing, Midwifery & Palliative Care, King’s College London, London, UK; 8 University of Nottingham, Nottingham, UK

**Keywords:** Health Workforce, COVID-19, Health Services, Job Satisfaction, MENTAL HEALTH

## Abstract

**Abstract:**

**Objectives:**

This study aims to determine key workforce variables (demographic, health and occupational) that predicted National Health Service (NHS) staff’s absence due to illness and expressed intention to leave their current profession.

**Design, setting and participants:**

Staff from 18 NHS Trusts were surveyed between April 2020 and January 2021, and again approximately 12 months later.

**Outcome measures:**

Logistic and linear regression were used to explore relationships between baseline exposures and four 12-month outcomes: absence due to COVID-19, absence due to non-COVID-19 illness, actively seeking employment outside current profession and regularly thinking about leaving current profession.

**Results:**

22 555 participants (out of a possible 152 286 employees; 15%) completed the baseline questionnaire. 10 831 participants completed the short follow-up questionnaire at 12 months and 5868 also completed the long questionnaire; these participants were included in the analyses of sickness absence and intention to leave, respectively. 20% of participants took 5+ days of work absence for non-COVID-19 sickness in the 12 months between baseline and 12-month questionnaire; 14% took 5+ days of COVID-19-related sickness absence. At 12 months, 20% agreed or strongly agreed they were actively seeking employment outside their current profession; 24% thought about leaving their profession at least several times per week. Sickness absence (COVID-19 and non-COVID-19 related) and intention to leave the profession (actively seeking another role and thinking about leaving) were all more common among NHS staff who were younger, in a COVID-19 risk group, had a probable mental health disorder, and who did not feel supported by colleagues and managers.

**Conclusions:**

Several factors affected both workforce retention and sickness absence. Of particular interest are the impact of colleague and manager support because they are modifiable. The NHS workforce is likely to benefit from training managers to speak with and support staff, especially those experiencing mental health difficulties. Further, staff should be given sufficient opportunities to form and foster social connections. Selection bias may have affected the presented results.

**STRENGTHS AND LIMITATIONS OF THIS STUDY**

- This study has a large sample size, with participants from 18 acute and mental health National Health Service (NHS) Trusts across England, in differing areas of deprivation, with a mixture of urban and rural settings, and a response rate of 15%.
- The data are longitudinal in nature, allowing us to explore predictive factors in a robust way, which is not possible with cross-sectional data used by most research in this area.
- We present data on intention to leave the profession and sickness absence, two major factors involved in the current NHS workforce crisis.
- Around half of the participants who completed the baseline questionnaire did not complete the 12-month follow-up questionnaire, and there was further missingness within the second part of the 12-month questionnaire.
- This study was carried out during the COVID-19 pandemic and, therefore, the proportions of people taking sick leave and intending to leave the NHS may differ from now; however, we believe our findings are still likely to be generalisable to the NHS workforce outside of the pandemic.

## Introduction

 The National Health Service (NHS; a government-funded universal healthcare provider in the UK), and in particular its staffing, is a regular topic of UK media and political debate. The reporting of this topic frequently states that the ability of the NHS to provide a good service in a timely manner is under more pressure and strain than ever before.^1^

There are many potential reasons for the current workforce difficulties faced by the NHS. These include the disruption caused by the COVID-19 pandemic and ongoing economic difficulties facing the UK.^2^ Sickness absence rates are reported to be at an all-time high,^3^,^5^ as are the number of staff intending to leave or leaving the NHS.^6^ Poor staffing levels mean remaining staff have more work to do, with 71% of NHS staff surveyed stating that they do not have as much time with patients as they would like.^7^ This can be a source of workplace stress which can become sickness absence; the Health and Safety Executive reports that, across all sectors, mental ill health is the most common reason for sickness absence from work.^8 9^ The reduction of sickness and absence is a key ambition of the long-term NHS Workforce plan.^10^

The NHS CHECK study was developed early in the pandemic to try to understand the mental health and well-being of clinical and non-clinical NHS healthcare workers across 18 Trusts in England.^11^ Data were collected early in the pandemic, and again 6, 12 and 24 months after the baseline questionnaire.^11^ While the original focus of these questionnaires was on the mental health implications of the pandemic for NHS staff, the data provides an opportunity to assess a wider range of aspects regarding the well-being of staff working in the NHS.

The aim of this study was to determine key baseline workforce variables (demographic, health and occupational) that predicted (1) NHS staff absence due to illness (both COVID-19 and non-COVID-19 related) and (2) the expressed intention to leave their current profession, 12 months later.

## Methods

### Setting and participants

18 English NHS Trusts were selected for this study, chosen for diversity in geographical location, urban and rural settings, and acute and mental health Trusts.^11^ Details of the 18 NHS Trusts are provided in the . All Trust staff were eligible to participate, including clinical (eg, doctors, nurses) and non-clinical (eg, administration roles, human resources roles, porters and cleaners) staff and staff on any contract type. Trusts were invited to participate via direct emails to senior leadership teams, building on the research team’s professional network.

### Data collection

Once recruited, Trusts invited all staff to participate in an online survey. The study was promoted via several routes, and both individuals and Trusts were incentivised with prizes.^11^ Participants provided online informed consent before starting the online survey. The baseline data collection period was April 2020 to January 2021. Follow-up data were collected approximately 6 and 12 months after each individual completed the baseline survey. 12-month data collection was completed between May 2021 and February 2022.^11^ 24-month data have since been added.

This paper focuses on baseline exposures and 12-month outcomes and only includes participants who completed, at least in part, the baseline and 12-month questionnaires. Questionnaires were split into two parts; participants completed the first part (‘short questionnaire’), then were asked if they wished to complete further questions in the second part (‘long questionnaire’). This was to encourage staff to participate even if they were short of time; 54% of participants who completed the short 12-month questionnaire also completed the long questionnaire. The intention to leave outcomes (and the COVID-19 risk group exposure variable) was recorded in the long questionnaires, and therefore, analyses of these outcomes have a small participant population.

### Outcomes

The four outcomes were responses to the following statements and questions, recorded in the 12-month questionnaire:

- ‘I am actively seeking employment outside my current profession or occupation’ (Strongly disagree, disagree, neither agree or disagree, agree, strongly agree). Dichotomised as agree/strongly agree versus the other three categories.
- ‘How often do you think about leaving your current profession or occupation?’ (Never, several times a year, several times a month, several times a week, everyday). Dichotomised as several times a month/week/daily versus the other two categories.
- ‘How many days in total in the last 12 months have you been absent from work due to ill health that is not related to COVID-19 (it is fine to estimate)?’
- ‘How many days in total have you been absent from work due to COVID-19 (it’s fine to estimate)?’

### Exposure variables

The exposure variables of interest were recorded in the baseline questionnaire and were selected a priori based on expert opinion:

- Age (categorised as ≤30, 31–40, 41–50, 51–60 and ≥61 years).
- Gender (male, female, other, prefer not to say).
- Ethnicity (white, black/African/Caribbean, Asian, mixed/multiple, other).
- Clinical role (doctor, nurse/midwife, other clinical, non-clinical).
- COVID-19 risk group membership (yes, no; defined by the question ‘Do you consider yourself as being in an at-risk group’ (eg, elderly, existing health conditions or pregnancy) for COVID-19 (coronavirus)?)
- Mental health status according to the General Health Questionnaire-12^12^ (GHQ-12; no common mental health disorder (GHQ-12<4), probable common mental health disorder (GHQ-12≥4)).
- Trust type (acute or mental health).
- Redeployed outside usual role (yes, no).
- Felt supported by colleagues (not at all, a little bit, moderately, quite a bit, extremely).
- Felt supported by manager (not at all, a little bit, moderately, quite a bit, extremely).

In addition, a covariable for the season in which the 12-month questionnaire was completed was included in all models, to account for seasonal differences in hospital admissions and, therefore, staff workload (spring/summer (May–August 2021), autumn (September–November 2021), winter (December 2021–February 2022)).

For the GHQ-12, the robustness of the measure has been established even where response options differ slightly from the original, as was the case in this study where there was a small typographical error in one response option of one GHQ-12 item.^13^ If a participant had completed 10 or 11 out of the 12 questions (n=296, 2.7%), then the most common score across the cohort for the given missing question was imputed for the remaining one or two questions. If only 1–9 questions (n=44, 0.4%) or zero questions (n=302, 2.8%) were completed, then no imputation was made (and the overall score could not be calculated). The overall score was calculated in the standard way.^12 14^ This overall score (min=0, max=12) was dichotomised according to standard guidelines as <4 (no mental health disorder) and ≥4 (probably common mental health disorder).

### Statistical analysis

Continuous data are summarised using means and SDs, or medians and IQRs if distributions are highly skewed. Categorical data are summarised as numbers and percentages.

Predictors of intention to leave the current profession were explored using logistic regression. We performed two sets of statistical models: one for actively seeking employment outside their current profession and one for thinking about leaving their current profession. Each exposure variable was fitted in the models in turn, adjusting for age, gender, ethnicity and season of 12-month questionnaire completion, as fixed effects. Odds ratios (ORs), 95% confidence intervals (CIs) and p values are presented.

Due to the large number of participants with no sick days, predictors of the number of non-COVID-19-related and COVID-19-related sick days were explored using two-part modelling. For each of the outcomes, logistic regression models were fitted with the outcome of any versus no sick days, and linear regression models were fitted only for the participants who had at least one sick day. These were fitted using the ‘twopm’ command in Stata, with the ‘suest’ option to combine the estimation results of the two parts of the model to derive a simultaneous variance matrix. Models were fitted for each exposure variable adjusting for demographic variables and season, as above. For the logistic regression parts, ORs, 95% CIs and p values are presented. Model fit of linear models was explored graphically, and the continuous part of all outcome variables was log transformed for best fit; estimates are presented as geometric mean ratios (GMRs), 95% CIs and p values.

For categorical variables with more than two categories, p values for the overall effect of the variable are also presented. Due to small numbers, mixed and other ethnicity categories were combined, and participants whose gender was other or prefer not to say were excluded in all models.

Analyses are all based on complete case data. To explore the effects of missing data, we present baseline characteristics for the full baseline cohort, as well as those who completed the short and long parts of the 12-month questionnaire in table 1, and present those with complete outcome data for each of the four outcomes in online supplemental table S1. Further, we explored which exposure variables predicted missingness at 12 months, taking the whole baseline cohort and predicting missingness in each of the four specific outcomes. These were modelled using logistic regression, adjusted the same as the outcome models. Findings from these analyses are presented in online supplemental table S2 and discussed in the limitations section in the discussion.

**Table 1:** Participant baseline characteristics

	Whole cohort(n=22 555)		12-month cohort-short questionnaire(n=10 831)		12-month cohort-long questionnaire(n=5868)	
	n	%	n	%	n	%
Age (years; median, IQR)	43	(33, 53)	47	(36, 54)	47	(36, 54)
≤30 years	4297	19.1	1494	13.8	793	13.5
31–40 years	4944	21.9	2034	18.8	1097	18.7
41–50 years	5638	25.0	2913	26.9	1633	27.8
51–60 years	5287	23.4	3104	28.7	1656	28.2
≥61 years	1333	5.9	804	7.4	461	7.9
Missing	1056	4.7	482	4.5	228	3.9
Gender						
Female	18 125	80.4	8785	81.1	4782	81.5
Male	4177	18.5	1964	18.1	1049	17.9
Other/prefer not to say	117	0.5	55	0.5	22	0.4
Missing	136	0.6	27	0.2	15	0.3
Ethnicity						
White	19 171	85.0	9640	89.0	5381	91.7
Black	973	4.3	337	3.1	126	2.1
Asian	1473	6.5	488	4.5	190	3.2
Mixed	546	2.4	241	2.2	107	1.8
Other	209	0.9	76	0.7	38	0.6
Missing	183	0.8	49	0.5	26	0.4
Clinical role						
Doctor	1634	7.2	708	6.5	377	6.4
Nurse/midwife	5726	25.4	2726	25.2	1517	25.9
Other clinical	6638	29.4	2998	27.7	1590	27.1
Non-clinical	8434	37.4	4372	40.4	2369	40.4
Missing	123	0.5	27	0.2	15	0.3
COVID-19 risk group*						
No	9170	40.7	5108	47.2	3218	54.8
Yes	3172	14.1	1882	17.4	1117	19.0
Missing	10 213	45.3	3841	35.5	1533	26.1
Mental health (MH) status						
No MH disorder (GHQ-12<4)	10 121	44.9	4923	45.5	2683	45.7
Probable MH disorder (GHQ-12≥4)	11 317	50.2	5562	51.4	3049	52.0
Missing	1117	5.0	346	3.2	136	2.3
Type of trust staff work for						
Acute	11 301	50.1	5582	51.5	3095	52.7
Mental health	11 254	49.9	5249	48.5	2773	47.3
Redeployed outside usual role						
No	19 442	86.2	9516	87.9	5179	88.3
Yes	2777	12.3	1213	11.2	646	11.0
Missing	336	1.5	102	0.9	43	0.7
Felt supported by colleagues						
Extremely	8368	37.1	4101	37.9	2286	39.0
Quite a bit	8217	36.4	3997	36.9	2138	36.4
Moderately	3450	15.3	1634	15.1	871	14.8
A little bit	1384	6.1	665	6.1	361	6.2
Not at all	313	1.4	158	1.5	91	1.6
Missing	823	3.6	276	2.5	121	2.1
Felt supported by manager						
Extremely	7077	31.4	3454	31.9	1894	32.3
Quite a bit	7061	31.3	3418	31.6	1835	31.3
Moderately	4021	17.8	1929	17.8	1030	17.6
A little bit	2396	10.6	1175	10.8	662	11.3
Not at all	1152	5.1	568	5.2	319	5.4
Missing	848	3.8	287	2.6	128	2.2

* In the 12-month short questionnaire cohort, this includes 13% with existing health conditions, 2% who identify as elderly, <1% who were pregnant and 3% for other reasons (categories not mutually exclusive).

GHQ, General Health Questionnaire; IQR, Interquartile range.

All data management and analyses were performed in Stata V.17.0.

### Patient and public involvement

The NHS CHECK survey was developed with input from a Patient and Public Involvement (PPI) advisory group. The acceptability of the questions, materials and data collection procedures were tested with this small reference group of front-line staff (psychologists, managers, administrators, security staff, intensivists and trainees) and the survey was refined accordingly.

## Results

### Baseline data

**Figure 1:**
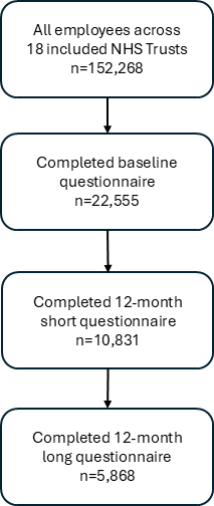
Participant flow chart. NHS, National Health Service.

Valid responses to the baseline questionnaire (collected April 2020–January 2021) were received from 22 555 participants, a 15% response rate from a potential denominator of 152 268 employees (figure 1). After excluding participants who did not complete the 12-month questionnaire, we included 10 831 participants for analysis (of these, 5868 completed the long questionnaire and therefore had the opportunity to complete the intention to leave outcome questions; figure 1). The median age of participants was 47 years (IQR 36–54), 81% were female and 89% were white. 60% of participants held clinical roles, comprising 7% doctors, 25% nurses/midwives and 28% other clinical roles. Demographics were similar between those who completed the baseline questionnaire and the short and long 12-month questionnaires (table 1).

### 12-month data

20% of participants (1169/5868) stated they agreed or strongly agreed that they were actively seeking employment outside their current profession; 43% (2528/5868) strongly disagreed with the statement (table 2). 30% of participants (1760/5868) stated they thought about leaving their profession either weekly or monthly, and a further 12% (731/5868) stated they thought about it every day (table 2).

**Table 2:** Intention to leave the profession and sickness absence at 12 months

Intention to leave current profession	12-month cohort-long questionnaire (n=5868)	
	n	%
I am actively seeking employment outside my current profession or occupation		
Strongly disagree	2528	43.1
Disagree	1067	18.2
Neither agree or disagree	1052	17.9
Agree	710	12.1
Strongly agree	459	7.8
Missing	52	0.9
How often do you think about leaving your current profession or occupation?		
Never	1631	27.8
Yearly—several times per year	1694	28.9
Monthly—several times per month	1044	17.8
Weekly—several times per week	726	12.4
Daily	731	12.5
Missing	42	0.7

Absence due to illness	12 month cohort-short questionnaire (n=10 831)	
	n	%
Days absent in past 12 months due to illness not related to COVID-19 (median, IQR)	0	(0, 5)
0 days	4221	39.0
1–4 days	2001	18.5
5–9 days	746	6.9
10–14 days	349	3.2
15+ days	1041	9.6
Missing	2473	22.8
Days absent due to COVID-19 (median, IQR)	0	(0, 0)
0 days	6581	60.8
1–4 days	170	1.6
5–9 days	339	3.1
10–14 days	556	5.1
15+ days	588	5.4
Missing	2597	24.0

39% of participants (4221/10 831) took no absence from work due to non-COVID-19 illness in the 12-month period before completing the questionnaire, 18% (2001/10 831) took 1–4 days, 10% (1095/10 831) took 5–14 days and 10% (1041/10 831) took 15days or more (table 2). 61% of participants (6581/10 831) had no work absence due to COVID-19, 2% (170/10 831) took 1–4 days of COVID-19-related absence, 8% (895/10 831) took 5–14 days and 5% (558/10 831) 15+ days (table 2).

### Actively seeking employment outside current profession

Factors associated with participants reporting actively seeking employment outside their current profession were: male gender (OR 1.18, 95% CI 1.00 to 1.40), being in a COVID-19 risk group (OR 1.34, 95% CI 1.12 to 1.59), having a probable mental health disorder (OR 1.89, 95% CI 1.64 to 2.18) and having less colleague (p<0.001) and manager (p<0.001) support (table 3). Older participants were less likely to be seeking employment outside their current profession (p=0.023). Clinical role was associated (p<0.001), such that doctors were less likely than non-clinical participants to be seeking employment outside their current profession (OR 0.38, 95% CI 0.27 to 0.55; table 3).

**Table 3:** Actively seeking a job outside my current profession or occupation (strongly agree/agree vs neither agree or disagree/disagree/strongly disagree)

Baseline predictor	Number with outcome		Adjusted analysis model		P value
	n	%	OR	95% CI	
Age					0.023
≤30 years	177	22.5	1		
31–40 years	237	21.8	0.96	0.77 to 1.20	0.721
41–50 years	326	20.2	0.87	0.71 to 1.07	0.180
51–60 years	300	18.4	0.78	0.63 to 0.97	0.022
61+ years	73	16.3	0.67	0.49 to 0.90	0.009
Gender					
Female	889	19.5	1		
Male	224	22.2	1.18	1.00 to 1.40	0.048
Ethnicity					0.097
White	1005	19.6	1		
Black/African/Caribbean	31	26.3	1.45	0.95 to 2.20	0.082
Asian	49	26.5	1.38	0.98 to 1.93	0.062
Mixed/multiple/other	28	20.1	0.98	0.64 to 1.50	0.933
Clinical role					<0.001
Non-clinical	470	21.1	1		
Doctor	37	10.2	0.38	0.27 to 0.55	<0.001
Nurse/midwife	314	21.6	1.03	0.87 to 1.21	0.750
Other clinical	290	19.1	0.85	0.72 to 1.01	0.064
COVID-19 risk group					
No	607	19.8	1		
Yes	253	23.9	1.34	1.12 to 1.59	0.001
Mental health (MH) status					
No MH disorder (GHQ-12<4)	374	14.7	1		
Probable MH disorder (GHQ-12≥4)	718	24.6	1.89	1.64 to 2.18	<0.001
Type of trust staff work for					
Acute	593	20.3	1		
Mental health	520	19.7	0.97	0.85 to 1.12	0.687
Redeployed outside usual role					
No	964	19.6	1		
Yes	144	23.2	1.21	0.99 to 1.48	0.061
Felt supported by colleagues					<0.001
Extremely	331	15.3	1		
Quite a lot	413	20.1	1.37	1.17 to 1.61	<0.001
Moderately	204	24.5	1.77	1.45 to 2.15	<0.001
A little	117	34.2	2.86	2.22 to 3.68	<0.001
Not at all	31	37.3	3.19	2.01 to 5.07	<0.001
Felt supported by manager					<0.001
Extremely	254	14.2	1		
Quite a lot	306	17.4	1.26	1.05 to 1.51	0.014
Moderately	226	23.1	1.80	1.47 to 2.20	<0.001
A little	198	31.3	2.76	2.23 to 3.42	<0.001
Not at all	112	37.1	3.58	2.73 to 4.69	<0.001

All analyses are adjusted for age, gender and ethnicity. Numbers and analyses only include patients with complete baseline age, gender and ethnicity data, complete outcome data and who completed the long questionnaire.

CI, confidence interval; GHQ-12, General Health Questionnaire 12; OR, odds ratio.

### Regularly thinking about leaving current profession

Clinical role (p<0.001; in particular being a nurse/midwife compared with a non-clinical role (OR 1.54, 95% CI 1.35 to 1.75)), being in a COVID-19 risk group (OR 1.19, 95% CI 1.02 to 1.37), having a probable mental health disorder (OR 2.28, 95% CI 2.04 to 2.55) and less colleague (p<0.001) and manager support (p<0.001) increased the odds of regularly thinking about leaving their current profession (table 4). Older participants were less likely to regularly think about leaving their role than younger participants (p=0.001; table 4).

**Table 4:** Thinking about leaving your current profession or occupation (every day/several times a week/ several times a month vs several times a year/never)

Baseline predictor	Number with outcome		Adjusted analysis model		P value
	n	%	OR	95% CI	
Age					<0.001
≤30 years	381	48.4	1		
31–40 years	466	42.9	0.80	0.67 to 0.97	0.021
41–50 years	643	39.7	0.70	0.59 to 0.83	<0.001
51–60 years	716	43.8	0.83	0.70 to 0.98	0.032
61+ years	180	39.7	0.70	0.55 to 0.89	0.003
Gender					
Female	1960	42.9	1		
Male	426	42.1	0.99	0.86 to 1.14	0.873
Ethnicity					0.360
White	2196	42.8	1		
Black/African/Caribbean	55	46.6	1.16	0.81 to 1.68	0.421
Asian	71	38.4	0.81	0.60 to 1.10	0.171
Mixed/multiple/other	64	46.4	1.14	0.81 to 1.61	0.447
Clinical role					<0.001
Non-clinical	873	39.1	1		
Doctor	153	42.3	1.17	0.93 to 1.47	0.187
Nurse/midwife	724	49.7	1.53	1.34 to 1.75	<0.001
Other clinical	634	41.7	1.10	0.96 to 1.25	0.187
COVID-19 risk group					
No	1320	43.0	1		
Yes	494	46.6	1.19	1.02 to 1.37	0.022
Mental health (MH) status					
No MH disorder (GHQ-12<4)	821	32.3	1		
Probable MH disorder (GHQ-12≥4)	1518	52.0	2.28	2.04 to 2.55	<0.001
Type of trust staff work for					
Acute	1287	43.9	1		
Mental health	1099	41.5	0.92	0.83 to 1.03	0.154
Redeployed outside usual role					
No	2097	42.6	1		
Yes	272	43.7	1.03	0.87 to 1.22	0.756
Felt supported by colleagues					<0.001
Extremely	757	34.9	1		
Quite a lot	886	43.2	1.41	1.25 to 1.60	<0.001
Moderately	445	53.4	2.13	1.81 to 2.50	<0.001
A little	208	60.8	2.91	2.30 to 3.68	<0.001
Not at all	50	61.0	2.98	1.90 to 4.70	<0.001
Felt supported by manager					<0.001
Extremely	568	31.6	1		
Quite a lot	680	38.6	1.36	1.18 to 1.56	<0.001
Moderately	500	51.1	2.26	1.92 to 2.65	<0.001
A little	386	61.0	3.37	2.79 to 4.06	<0.001
Not at all	207	68.8	4.75	3.65 to 6.19	<0.001

All analyses are adjusted for age, gender and ethnicity. Numbers and analyses only include patients with complete baseline age, gender and ethnicity data, complete outcome data and who completed the long questionnaire.

CI, confidence interval; GHQ-12, General Health Questionnaire 12; OR, odds ratio.

### Non-COVID-19-related sick leave

Older participants had lower odds of taking non-COVID-19-related sick leave than younger participants (p<0.001), but if they took it, it was slightly longer (p<0.001; table 5). Males had lower odds of taking non-COVID-19 sick leave (OR 0.73, 95% CI 0.65 to 0.82), but if they took it, their lengths of leave were similar to females (p=0.770). Doctors were less likely to take non-COVID-19 sick leave than non-clinical colleagues (OR 0.64, 95% CI 0.53 to 0.78), and where they did, it was shorter (GMR 0.76, 95% CI 0.62 to 0.93). Nurses/midwives and other clinical staff had higher odds of taking non-COVID-19 sick leave than non-clinical staff (OR 1.32 (95% CI 1.18 to 1.48) and OR 1.32 (95% CI 1.18 to 1.47), respectively) and were off sick for longer (table 5).

**Table 5:** Non-COVID-19-related sick days in previous 12 months

Non-COVID-19 sick days	Number with outcome		Dichotomised analyses of any sick days vs none			Continuous analyses of participants with at least 1 sick day reported		
Baseline predictor	n	%	OR	95% CI	P value	GMR	95% CI	P value
Age					<0.001			<0.001
≤30 years	647	60.5	1			1		
31–40 years	868	55.4	0.83	0.71 to 0.97	0.020	0.98	0.86 to 1.11	0.747
41–50 years	1091	47.2	0.59	0.51 to 0.68	<0.001	1.02	0.90 to 1.16	0.744
51–60 years	1102	45.4	0.55	0.47 to 0.63	<0.001	1.24	1.09 to 1.41	0.001
61+ years	255	40.7	0.46	0.37 to 0.56	<0.001	1.17	0.96 to 1.43	0.118
Gender								
Female	3357	51.0	1			1		
Male	606	42.7	0.73	0.65 to 0.82	<0.001	0.98	0.88 to 1.10	0.770
Ethnicity					0.307			0.656
White	3576	49.4	1			1		
Black/African/Caribbean	99	45.8	0.92	0.70 to 1.22	0.568	0.92	0.71 to 1.19	0.511
Asian	166	51.6	1.03	0.82 to 1.29	0.797	1.11	0.90 to 1.37	0.315
Mixed/multiple/other	122	56.2	1.29	0.98 to 1.69	0.073	1.07	0.84 to 1.35	0.594
Clinical role					<0.001			<0.001
Non-clinical	1473	45.7	1			1		
Doctor	183	34.5	0.64	0.53 to 0.78	<0.001	0.76	0.62 to 0.93	0.008
Nurse/midwife	1063	53.4	1.32	1.18 to 1.48	<0.001	1.20	1.08 to 1.34	0.001
Other clinical	1242	55.1	1.32	1.18 to 1.47	<0.001	1.11	1.00 to 1.23	0.048
COVID-19 risk group								
No	1884	48.2	1			1		
Yes	769	54.4	1.46	1.29 to 1.67	<0.001	1.43	1.27 to 1.62	<0.001
Mental health (MH) status								
No MH disorder (GHQ-12<4)	1576	43.4	1			1		
Probable MH disorder (GHQ-12≥4)	2293	54.9	1.54	1.41 to 1.69	<0.001	1.39	1.28 to 1.52	<0.001
Type of trust staff work for								
Acute	2008	49.5	1			1		
Mental health	1955	49.6	0.97	0.88 to 1.06	0.446	1.14	1.05 to 1.25	0.003
Redeployed outside usual role								
No	3483	49.3	1			1		
Yes	455	51.5	1.07	0.93 to 1.23	0.36	1.09	0.95 to 1.25	0.199
Felt supported by colleagues					<0.001			<0.001
Extremely	1438	47.1	1			1		
Quite a lot	1459	49.2	1.07	0.96 to 1.18	0.205	0.95	0.86 to 1.05	0.318
Moderately	654	53.4	1.25	1.09 to 1.43	0.001	1.20	1.05 to 1.37	0.006
A little	273	55.7	1.39	1.15 to 1.69	0.001	1.44	1.19 to 1.74	<0.001
Not at all	64	58.7	1.62	1.09 to 2.41	0.018	1.8	1.27 to 2.56	0.001
Felt supported by manager					<0.001			<0.001
Extremely	1195	46.8	1			1		
Quite a lot	1247	48.0	1.04	0.93 to 1.16	0.474	0.97	0.87 to 1.08	0.592
Moderately	737	52.1	1.21	1.06 to 1.38	0.004	1.10	0.97 to 1.25	0.156
A little	493	56.7	1.46	1.25 to 1.71	<0.001	1.14	0.98 to 1.31	0.089
Not at all	210	53.0	1.28	1.03 to 1.59	0.024	1.76	1.44 to 2.16	<0.001

All analyses are adjusted for age, gender and ethnicity. Numbers and analyses only include patients with complete baseline age, gender and ethnicity data, and complete outcome data.

CI, confidence interval; GHQ-12, General Health Questionnaire 12; GMR, geometric mean ratio; OR, odds ratio.

Participants in COVID-19 risk groups had higher odds of taking non-COVID-19 sick leave (OR 1.46, 95% CI 1.29 to 1.67) and if they did, it was also longer (GMR 1.43, 95% CI 1.27 to 1.62). Similarly, participants with a probable mental health disorder had higher odds of taking sick leave and for longer periods than participants with no mental health disorder (OR 1.54 (95% CI 1.41 to 1.69) and GMR 1.39 (95% CI 1.28 to 1.52), respectively). Participants working in mental health Trusts had similar odds of taking non-COVID-19 sick leave as those in acute Trusts (p=0.446), but if they did take it, it was slightly longer (GMR 1.14, 95% CI 1.05 to 1.25). Participants with less colleague (p<0.001) and manager (p<0.001) support tended to have higher odds of taking sick leave, and longer periods of sick leave (p<0.001), than participants with better support (table 5).

### COVID-19-related sick leave

Older participants had lower odds of taking COVID-19-related sick leave than younger participants (p<0.001), but where they did, it was for longer periods (p<0.001; table 6). All non-white participants had higher odds of COVID-19 sick leave than white participants (p<0.001), with participants of Asian ethnicity having the highest odds (OR 1.63, 95% CI 1.28 to 2.08). Asian participants also had longer periods of COVID-19 sick leave than white participants (GMR 1.37, 95% CI 1.11 to 1.69). Doctors, nurses/midwives and other clinical staff all had higher odds of taking COVID-19-related sick leave than non-clinical staff (p<0.001) and nurses/midwives and other clinical staff also had longer periods of COVID-19-related absence (p<0.001; table 6).

**Table 6:** COVID-19-related sick days

Baseline predictor	Number with outcome		Dichotomised analysis of any sick days vs none			Continuous analysis of participants with at least 1 sick day reported		
	n	%	OR	95% CI	P value	GMR	95% CI	P value
Age					<0.001			<0.001
≤30 years	245	23.9	1			1		
31–40 years	323	21.5	0.87	0.72 to 1.05	0.146	1.30	1.12 to 1.51	0.001
41–50 years	457	20.5	0.84	0.70 to 1.00	0.048	1.38	1.19 to 1.60	<0.001
51–60 years	476	19.5	0.79	0.66 to 0.94	0.008	1.44	1.25 to 1.67	<0.001
61+ years	71	11.0	0.41	0.31 to 0.55	<0.001	1.43	1.06 to 1.92	0.018
Gender								
Female	1284	19.9	1			1		
Male	288	20.7	1.05	0.91 to 1.21	0.527	0.98	0.88 to 1.10	0.759
Ethnicity					<0.001			0.017
White	1366	19.3	1			1		
Black/African/Caribbean	57	26.9	1.50	1.10 to 2.05	0.010	0.99	0.79 to 1.25	0.950
Asian	99	29.6	1.63	1.28 to 2.08	<0.001	1.37	1.11 to 1.69	0.003
Mixed/multiple/other	50	24.2	1.26	0.91 to 1.75	0.159	1.06	0.82 to 1.37	0.674
Clinical role					<0.001			<0.001
Non-clinical	489	15.4	1			1		
Doctor	119	23.2	1.54	1.22 to 1.95	<0.001	0.94	0.78 to 1.13	0.495
Nurse/midwife	466	23.4	1.62	1.41 to 1.87	<0.001	1.44	1.28 to 1.62	<0.001
Other clinical	496	22.9	1.55	1.34 to 1.78	<0.001	1.27	1.12 to 1.43	<0.001
COVID-19 risk group								
No	782	20.5	1			1		
Yes	265	19.1	0.99	0.84 to 1.16	0.885	1.59	1.38 to 1.84	<0.001
Mental health (MH) status								
No MH disorder (GHQ-12<4)	662	18.3	1			1		
Probable MH disorder (GHQ-12≥4)	882	21.9	1.22	1.09 to 1.37	0.001	1.29	1.17 to 1.42	<0.001
Type of trust staff work for								
Acute	891	22.2	1			1		
Mental health	681	17.8	0.75	0.67 to 0.84	<0.001	0.91	0.82 to 1.01	0.078
Redeployed outside usual role								
No	1380	19.9	1			1		
Yes	180	21.1	1.02	0.86 to 1.22	0.822	1.01	0.87 to 1.16	0.935
Felt supported by colleagues					0.372			0.707
Extremely	607	20.2	1			1		
Quite a lot	584	19.9	0.96	0.84 to 1.09	0.493	1.00	0.90 to 1.11	0.995
Moderately	254	21.7	1.05	0.89 to 1.24	0.576	1.04	0.91 to 1.20	0.568
A little	82	17.7	0.82	0.63 to 1.06	0.124	1.04	0.85 to 1.27	0.707
Not at all	19	17.4	0.79	0.47 to 1.33	0.375	1.34	0.75 to 2.41	0.323
Felt supported by manager					0.174			0.050
Extremely	479	18.8	1			1		
Quite a lot	500	19.7	1.05	0.91 to 1.21	0.486	0.99	0.88 to 1.11	0.844
Moderately	291	21.2	1.11	0.95 to 1.31	0.199	1.04	0.91 to 1.20	0.536
A little	183	21.5	1.13	0.93 to 1.37	0.23	1.20	1.03 to 1.40	0.023
Not at all	92	24.5	1.35	1.04 to 1.75	0.022	1.24	0.98 to 1.58	0.076

All analyses are adjusted for age, gender and ethnicity. Numbers and analyses only include patients with complete baseline age, gender and ethnicity data, and complete outcome data.

CI, confidence interval; GHQ-12, General Health Questionnaire 12; GMR, geometric mean ratio; OR, odds ratio.

The odds of COVID-19-related sick leave were not associated with COVID-19 risk group (p=0.885), but if they did take it, those in a high-risk group took longer leave (GMR 1.59, 95% CI 1.38 to 1.84). Participants with a probable mental health disorder had higher odds of taking COVID-19-related sick leave (OR 1.22, 95% CI 1.09 to 1.37) and had longer leave if they did take it (GMR 1.29, 95% CI 1.17 to 1.42). Participants working at a mental health Trust had lower odds of taking COVID-19-related sick leave than those at an acute Trust (OR 0.75, 95% CI 0.67 to 0.84), and slightly shorter periods of leave if they did take it (GMR 0.91, 95% CI 0.82 to 1.01). Manager support was not associated with taking COVID-19-related leave, but if it was taken, those who felt less supported took slightly longer leave (p=0.050; table 6).

## Discussion

### Summary of findings

This paper aimed to identify factors associated with staff intention to leave their NHS role and sickness absence. The profile of characteristics associated with these two outcomes was very similar; outcomes were more likely among NHS staff who were younger, in a COVID-19 risk group, had a probable mental health disorder, and who did not feel supported by colleagues and managers.

### Comparison to other literature

The association with age may be partly explained by ‘healthy worker survivor bias’, that is, healthier workers, and those willing and able to cope with the pressures inherent in the role, remain working (and are therefore in our cohort), while those who become unwell take more sickness absences and/or leave the workforce.^15^ Further, it seems likely that younger employees who are unhappy in their work would be more likely to seek alternative employment than older staff who may be contemplating retirement. Our findings about those in COVID-19 high risk groups are in line with the fact that the majority of high risk participants had a pre-existing health condition (eg, cardiovascular disease, diabetes or immune system diseases),^16^ making it more likely for them to take sickness absence and to consider leaving high-risk roles.^17^ There is a large body of evidence that those with poor mental health are more likely to take sickness absence and to exit the workforce,^8 9 18^ which is in keeping with our findings here. Our findings about those who feel unsupported by colleagues and managers are in line with other evidence that poorer perceived leadership and peer support are associated with both higher intention to leave^19^ and sickness absence.^20^ Similarly, a recent paper found that not feeling valued was associated with attrition from the UK healthcare workforce,^18^ and another stated that workforce stewardship should be a key NHS priority.^21^

Nurses/midwives were more likely than non-clinical staff to be thinking about leaving, while doctors (compared with non-clinical staff) and male participants were less likely to be actively seeking other roles. Given their long training, doctors may be less likely to seek other employment than non-clinical staff, who may perceive wider employment opportunities outside healthcare. The pressures inherent in nursing roles may be the reason they are more likely than non-clinical staff to consider leaving. The COVID-19 pandemic may have had a greater impact on women leaving the paid workforce due to their greater contribution to unpaid care work,^22^ which may explain our finding that male staff were less likely than female staff to be seeking a new role.

Female staff were more likely to take non-COVID-19 sickness absence, as were nurses/midwives or other clinical staff compared with non-clinical staff. Sickness absence rates have been historically higher for women,^23^ which is echoed in our findings. The fact that nurses/midwives and other clinical staff were more likely to take sickness absence may be due to the ability of some non-clinical staff to work from home, even when feeling unwell, whereas this is less feasible in clinical roles. In addition, high levels of sickness among nursing staff have been widely reported outside of the COVID-19 pandemic.^24 25^

In terms of COVID-19 sickness absence, staff of non-white ethnicities were more likely to be absent, as were those in clinical roles (compared with non-clinical roles), and those working in an acute hospital rather than mental health settings. This supports other evidence of higher risk of infection with COVID-19 and of worse clinical outcomes for people from minoritised ethnic backgrounds compared with white people.^26^ Acute hospital workers were more likely to be on site facing higher infection risks (so required more COVID-19 sickness absence) than those in mental health settings who were more likely to be able to move services to virtual settings.

Interestingly, we found a much lower rate of sickness absence (median 0 days (IQR 0–5) for non-COVID-19-related sickness absence and median 0 days (IQR 0 to 0) for COVID-19-related absence) than another cohort study in Greece looking at Health Care Professional (HCP) absence in vaccinated (mean 6.9 days, SD 5.7) and unvaccinated (mean 11.9 days, SD 8.1) workers over a 5-month period, November 2020–April 2021. This may have been due to participants under-reporting sick leave in our study, or perhaps simply higher rates of absence in Greece compared with the UK during this pandemic period.^27^

### Strengths and limitations of the study

The main strength of this study is the sample size, with participants from 18 acute and mental health NHS Trusts across England, in differing areas of deprivation, with a mixture of urban and rural settings, and a response rate of 15%. Another strength is the longitudinal nature of the data analysed. The availability of follow-up data has enabled us to explore predictive factors in a robust way, which is not possible with cross-sectional data used by most research in this area. We present data on both intention to leave and sickness absence, two major factors involved in the current NHS workforce crisis.^4^

However, there is some potential bias in our sample. Only 15% of those who were approached participated in the survey, increasing the likelihood of selection bias. However, we believe this response rate is higher than most surveys conducted during the pandemic. Further, around half of the participants (11 724/22 555; 52%) who completed the baseline questionnaire did not complete the 12-month follow-up questionnaire, and around half (4963/10 831) who completed the first part of the 12 month questionnaire did not complete the second part (which included the ‘intention to leave’ outcome questions). To explore this, we present the baseline characteristics for each of the cohorts of data (online supplemental table S1) and modelled which exposures were associated with missingness in each of the outcomes (online supplemental table S2). Findings were generally in the same direction as the effects in the outcome models, which, if anything, is likely to have led to an underestimation of associations between exposures and outcomes rather than an overestimation.

This study was carried out during the COVID-19 pandemic and therefore, given the heightened staff stress during that time and the large turnover of staff since then, the proportions of people taking sick leave and intending to leave the NHS may differ from now; however, we believe our findings are still likely to be generalisable to the NHS workforce outside of the pandemic.^28^ Unfortunately, we do not have prepandemic data for this cohort, so we are unable to provide insight into any changing trends from before these data were collected. A final limitation is that we did not collect vaccination status, so we were unable to assess its impact on sick leave.

### Implications for research and/or practice

Further research in this area could usefully explore methodological advances aimed at accounting for healthy worker survivor bias.^15 29^ The ability to link cohort data to prepandemic health and well-being, sickness absence and retention data should be explored, as this could offer insights into the long-term trajectories of these outcomes.

The clear stepwise association between how well supported someone feels by their colleagues and managers and the likelihood of actively thinking about job seeking outside their profession has important implications for employers. There are likely to be considerable benefits from training managers to speak with and support staff, especially those who are experiencing mental health difficulties. Furthermore, ensuring teams have sufficient opportunities to form and foster social connections and reflect on the challenges of their work together may reduce the likelihood of staff leaving their role or taking excessive sick days. This is already partially addressed in the recently published NHS long-term workforce plan.^10^

## Supplementary material

10.1136/bmjopen-2024-097483 online supplemental file 1

## Data Availability

Data are available upon reasonable request.
