# Supplementary material for: Predictors of sickness absence and intention to leave the profession among NHS staff in England during the COVID-19 pandemic: a prospective cohort study
Source: BMJ Open. 2025 Jun 4;15(6):e097483. doi: 10.1136/bmjopen-2024-097483 (PMC12142032; doi:10.1136/bmjopen-2024-097483)
Supplement: online supplemental file 1 [file bmjopen-15-6-s001.docx]

**Supplementary material**

**The 18 included NHS Trusts:**

Avon and Wiltshire Mental Health NHS Foundation Trust (n=4,334)

Cambridge University Hospitals NHS Foundation Trust (n=10,243)

Cambridgeshire and Peterborough NHS Foundation Trust (n=4,235)

Cornwall Partnership NHS Foundation Trust (n=3,977)

Devon Partnership NHS Foundation Trust (n=3,280)

East Suffolk and North Essex NHS Foundation Trust (n=10,219)

Gloucestershire Hospitals NHS Foundation Trust (n=8,437)

Guys and St Thomas" NHS Foundation Trust (n=19,760)

King’s College Hospital and Princess Royal University Hospital (PRUH) (n=12,959)

Lancashire and South Cumbria NHS Foundation Trust (n=6,984)

Norfolk and Norwich University Hospitals (n=10,502)

Nottinghamshire Healthcare NHS Foundation Trust (n=8,860)

Royal Papworth Hospital (n=2,110)

Sheffield Health and Social Care (n=2,610)

South London and Maudsley NHS Foundation Trust (n=5,151)

Tees Esk and Wear Valleys NHS Foundation Trust (n=7,315)

University Hospitals of Derby and Burton (n=13,231)

University Hospitals of Leicester NHS Foundation Trust (n=16,946).

Study sites were selected to cover multiple areas of England. All staff in each study site were invited to participate.

**Table S1 Participant baseline characteristics for participants who reported data for each of the four outcomes**

|  | | **Whole cohort**  **(n=22,438)** | | **12-month cohort**  **(n=10,776)** | | **Non-COVID sick leave (n=8,320)** | | **COVID sick leave**  **(n=8,198)** | | **Actively seeking new role (n=5,795)** | | **Thinking about leaving (n=5,805)** | |
| --- | --- | --- | --- | --- | --- | --- | --- | --- | --- | --- | --- | --- | --- |
|  | | **n** | **%** | **n** | **%** | **n** | **%** | **n** | **%** | **n** | **%** | **n** | **%** |
| **Age (median, IQR)** | | 43 | (33, 53) | 47 | (36, 54) | 47 | (37, 54) | 47 | (37, 55) | 47 | (36, 54) | 47 | (36, 54) |
|  | ≤30 years | 4273 | 19.0% | 1485 | 13.8% | 1070 | 12.9% | 1023 | 12.5% | 788 | 13.6% | 787 | 13.6% |
|  | 31-40 years | 4915 | 21.9% | 2019 | 18.7% | 1567 | 18.8% | 1503 | 18.3% | 1087 | 18.8% | 1087 | 18.7% |
|  | 41-50 years | 5620 | 25.0% | 2904 | 26.9% | 2311 | 27.8% | 2231 | 27.2% | 1617 | 27.9% | 1618 | 27.9% |
|  | 51-60 years | 5272 | 23.5% | 3097 | 28.7% | 2433 | 29.2% | 2450 | 29.9% | 1633 | 28.2% | 1638 | 28.2% |
|  | ≥61 years | 1328 | 5.9% | 802 | 7.4% | 626 | 7.5% | 646 | 7.9% | 449 | 7.7% | 453 | 7.8% |
|  | Missing | 1030 | 4.6% | 469 | 4.4% | 313 | 3.8% | 345 | 4.2% | 221 | 3.8% | 222 | 3.8% |
| **Gender** | |  |  |  |  |  |  |  |  |  |  |  |  |
|  | Female | 18125 | 80.8% | 8785 | 81.5% | 6839 | 82.2% | 6744 | 82.3% | 4737 | 81.7% | 4747 | 81.8% |
|  | Male | 4177 | 18.6% | 1964 | 18.2% | 1462 | 17.6% | 1437 | 17.5% | 1043 | 18.0% | 1043 | 18.0% |
|  | Missing | 136 | 0.6% | 27 | 0.3% | 19 | 0.2% | 17 | 0.2% | 15 | 0.3% | 15 | 0.3% |
| **Ethnicity** | |  |  |  |  |  |  |  |  |  |  |  |  |
|  | White | 19093 | 85.1% | 9604 | 89.1% | 7506 | 90.2% | 7381 | 90.0% | 5315 | 91.7% | 5326 | 91.7% |
|  | Black | 969 | 4.3% | 334 | 3.1% | 228 | 2.7% | 225 | 2.7% | 124 | 2.1% | 124 | 2.1% |
|  | Asian | 1465 | 6.5% | 484 | 4.5% | 334 | 4.0% | 351 | 4.3% | 190 | 3.3% | 190 | 3.3% |
|  | Mixed | 539 | 2.4% | 238 | 2.2% | 173 | 2.1% | 167 | 2.0% | 104 | 1.8% | 103 | 1.8% |
|  | Other | 199 | 0.9% | 72 | 0.7% | 51 | 0.6% | 48 | 0.6% | 38 | 0.7% | 38 | 0.7% |
|  | Missing | 173 | 0.8% | 44 | 0.4% | 28 | 0.3% | 26 | 0.3% | 24 | 0.4% | 24 | 0.4% |
| **Clinical role** | |  |  |  |  |  |  |  |  |  |  |  |  |
|  | Doctor | 1623 | 7.2% | 704 | 6.5% | 548 | 6.6% | 531 | 6.5% | 373 | 6.4% | 373 | 6.4% |
|  | Nurse/midwife | 5707 | 25.4% | 2717 | 25.2% | 2061 | 24.8% | 2077 | 25.3% | 1500 | 25.9% | 1502 | 25.9% |
|  | Other clinical | 6601 | 29.4% | 2983 | 27.7% | 2331 | 28.0% | 2256 | 27.5% | 1572 | 27.1% | 1576 | 27.1% |
|  | Non-clinical | 8384 | 37.4% | 4345 | 40.3% | 3360 | 40.4% | 3318 | 40.5% | 2335 | 40.3% | 2339 | 40.3% |
|  | Missing | 123 | 0.5% | 27 | 0.3% | 20 | 0.2% | 16 | 0.2% | 15 | 0.3% | 15 | 0.3% |
| **Covid risk group** | |  |  |  |  |  |  |  |  |  |  |  |  |
|  | No | 9124 | 40.7% | 5082 | 47.2% | 4052 | 48.7% | 3969 | 48.4% | 3178 | 54.8% | 3182 | 54.8% |
|  | Yes | 3161 | 14.1% | 1875 | 17.4% | 1473 | 17.7% | 1454 | 17.7% | 1104 | 19.1% | 1104 | 19.0% |
|  | Missing | 10153 | 45.2% | 3819 | 35.4% | 2795 | 33.6% | 2775 | 33.8% | 1513 | 26.1% | 1519 | 26.2% |
| **Mental health (MH) status** | |  |  |  |  |  |  |  |  |  |  |  |  |
|  | No MH disorder (GHQ12<4) | 10075 | 44.9% | 4903 | 45.5% | 3781 | 45.4% | 3790 | 46.2% | 2643 | 45.6% | 2651 | 45.7% |
|  | Probable MH disorder (GHQ12≥4) | 11254 | 50.2% | 5529 | 51.3% | 4311 | 51.8% | 4184 | 51.0% | 3018 | 52.1% | 3020 | 52.0% |
|  | Missing | 1109 | 4.9% | 344 | 3.2% | 228 | 2.7% | 224 | 2.7% | 134 | 2.3% | 134 | 2.3% |
| **Trust type** | |  |  |  |  |  |  |  |  |  |  |  |  |
|  | Acute trust | 11241 | 50.1% | 5549 | 51.5% | 4244 | 51.0% | 4228 | 51.6% | 3065 | 52.9% | 3073 | 52.9% |
|  | Mental Health trust | 11197 | 49.9% | 5227 | 48.5% | 4076 | 49.0% | 3970 | 48.4% | 2730 | 47.1% | 2732 | 47.1% |
| **Redeployed outside usual role** | |  |  |  |  |  |  |  |  |  |  |  |  |
|  | No | 19340 | 86.2% | 9468 | 87.9% | 7345 | 88.3% | 7248 | 88.4% | 5116 | 88.3% | 5125 | 88.3% |
|  | Yes | 2765 | 12.3% | 1207 | 11.2% | 910 | 10.9% | 886 | 10.8% | 637 | 11.0% | 637 | 11.0% |
|  | Missing | 333 | 1.5% | 101 | 0.9% | 65 | 0.8% | 64 | 0.8% | 42 | 0.7% | 43 | 0.7% |
| **Supported by colleagues** | |  |  |  |  |  |  |  |  |  |  |  |  |
|  | Extremely | 8334 | 37.1% | 4086 | 37.9% | 3179 | 38.2% | 3159 | 38.5% | 2258 | 39.0% | 2267 | 39.1% |
|  | Quite a bit | 8189 | 36.5% | 3984 | 37.0% | 3075 | 37.0% | 3045 | 37.1% | 2120 | 36.6% | 2120 | 36.5% |
|  | Moderately | 3420 | 15.2% | 1618 | 15.0% | 1258 | 15.1% | 1209 | 14.7% | 859 | 14.8% | 860 | 14.8% |
|  | A little bit | 1367 | 6.1% | 657 | 6.1% | 507 | 6.1% | 485 | 5.9% | 353 | 6.1% | 353 | 6.1% |
|  | Not at all | 311 | 1.4% | 157 | 1.5% | 116 | 1.4% | 115 | 1.4% | 90 | 1.6% | 89 | 1.5% |
|  | Missing | 817 | 3.6% | 274 | 2.5% | 185 | 2.2% | 185 | 2.3% | 115 | 2.0% | 116 | 2.0% |
| **Supported by manager** | |  |  |  |  |  |  |  |  |  |  |  |  |
|  | Extremely | 7052 | 31.4% | 3443 | 32.0% | 2670 | 32.1% | 2668 | 32.5% | 1872 | 32.3% | 1878 | 32.4% |
|  | Quite a bit | 7040 | 31.4% | 3409 | 31.6% | 2681 | 32.2% | 2635 | 32.1% | 1814 | 31.3% | 1816 | 31.3% |
|  | Moderately | 3989 | 17.8% | 1912 | 17.7% | 1467 | 17.6% | 1424 | 17.4% | 1015 | 17.5% | 1017 | 17.5% |
|  | A little bit | 2374 | 10.6% | 1164 | 10.8% | 897 | 10.8% | 883 | 10.8% | 653 | 11.3% | 653 | 11.2% |
|  | Not at all | 1141 | 5.1% | 563 | 5.2% | 411 | 4.9% | 395 | 4.8% | 318 | 5.5% | 317 | 5.5% |
|  | Missing | 842 | 3.8% | 285 | 2.6% | 194 | 2.3% | 193 | 2.4% | 123 | 2.1% | 124 | 2.1% |

IQR=Interquartile range. GHQ12=General health questionnaire. Note. Participants who reported their gender as other or prefer not to say have been excluded from this table as numbers were too small to include in analysis models.

**Table S2 Predicting missingness of job hunt and sick leave questions in the 12-month questionnaire**

|  | | Actively seeking a new job | | | Regularly thinking about leaving profession | | | Non-COVID-19 sick leave | | | COVID-19 sick leave | | |
| --- | --- | --- | --- | --- | --- | --- | --- | --- | --- | --- | --- | --- | --- |
| Baseline predictor | | OR | 95% CI | P-value | OR | 95% CI | P-value | OR | 95% CI | P-value | OR | 95% CI | P-value |
| Age: | |  |  | <0.001 |  |  | <0.001 |  |  | <0.001 |  |  | <0.001 |
|  | ≤30 years | 1 |  |  | 1 |  |  | 1 |  |  | 1 |  |  |
|  | 31-40 years | 0.81 | 0.73 to 0.90 | <0.001 | 0.81 | 0.73 to 0.90 | <0.001 | 0.72 | 0.66 to 0.79 | <0.001 | 0.72 | 0.66 to 0.79 | <0.001 |
|  | 41-50 years | 0.57 | 0.52 to 0.63 | <0.001 | 0.57 | 0.52 to 0.63 | <0.001 | 0.49 | 0.44 to 0.53 | <0.001 | 0.48 | 0.44 to 0.53 | <0.001 |
|  | 51-60 years | 0.54 | 0.49 to 0.59 | <0.001 | 0.53 | 0.48 to 0.59 | <0.001 | 0.41 | 0.37 to 0.45 | <0.001 | 0.38 | 0.34 to 0.41 | <0.001 |
|  | 61+ years | 0.47 | 0.41 to 0.54 | <0.001 | 0.47 | 0.41 to 0.54 | <0.001 | 0.39 | 0.35 to 0.45 | <0.001 | 0.35 | 0.30 to 0.39 | <0.001 |
| Gender: | |  |  |  |  |  |  |  |  |  |  |  |  |
|  | Female | 1 |  |  | 1 |  |  | 1 |  |  | 1 |  |  |
|  | Male | 1.03 | 0.96 to 1.12 | 0.407 | 1.04 | 0.96 to 1.12 | 0.367 | 1.12 | 1.04 to 1.20 | 0.003 | 1.13 | 1.05 to 1.21 | 0.001 |
| Ethnicity: | |  |  | <0.001 |  |  | <0.001 |  |  | <0.001 |  |  | <0.001 |
|  | White | 1 |  |  | 1 |  |  | 1 |  |  | 1 |  |  |
|  | Black/African/Caribbean | 2.60 | 2.13 to 3.16 | <0.001 | 2.60 | 2.14 to 3.17 | <0.001 | 2.09 | 1.78 to 2.44 | <0.001 | 2.06 | 1.76 to 2.41 | <0.001 |
|  | Asian | 2.25 | 1.91 to 2.63 | <0.001 | 2.25 | 1.92 to 2.64 | <0.001 | 1.84 | 1.61 to 2.09 | <0.001 | 1.66 | 1.46 to 1.89 | <0.001 |
|  | Mixed/multiple/other | 1.50 | 1.24 to 1.81 | <0.001 | 1.51 | 1.25 to 1.83 | <0.001 | 1.36 | 1.15 to 1.60 | <0.001 | 1.39 | 1.17 to 1.64 | <0.001 |
| Clinical role: | |  |  | 0.011 |  |  | 0.013 |  |  | 0.014 |  |  | 0.015 |
|  | Non-clinical | 1 |  |  | 1 |  |  | 1 |  |  | 1 |  |  |
|  | Doctor | 1.10 | 0.96 to 1.25 | 0.177 | 1.10 | 0.96 to 1.25 | 0.173 | 1.10 | 0.98 to 1.24 | 0.105 | 1.13 | 1.01 to 1.28 | 0.039 |
|  | Nurse/midwife | 1.01 | 0.93 to 1.09 | 0.871 | 1.01 | 0.93 to 1.09 | 0.852 | 1.11 | 1.04 to 1.20 | 0.004 | 1.07 | 1.00 to 1.15 | 0.062 |
|  | Other clinical | 1.13 | 1.04 to 1.22 | 0.003 | 1.12 | 1.04 to 1.22 | 0.003 | 1.09 | 1.02 to 1.17 | 0.018 | 1.11 | 1.03 to 1.19 | 0.004 |
| COVID risk group: | |  |  |  |  |  |  |  |  |  |  |  |  |
|  | No | 1 |  |  | 1 |  |  | 1 |  |  | 1 |  |  |
|  | Yes | 1.06 | 0.97 to 1.16 | 0.214 | 1.06 | 0.97 to 1.16 | 0.190 | 1.00 | 0.92 to 1.09 | 0.981 | 1.01 | 0.93 to 1.11 | 0.780 |
| Mental health (MH) status: | |  |  |  |  |  |  |  |  |  |  |  |  |
|  | No MH disorder (GHQ12<4) | 1 |  |  | 1 |  |  | 1 |  |  | 1 |  |  |
|  | Probable MH disorder (GHQ12≥4) | 0.95 | 0.89 to 1.01 | 0.123 | 0.96 | 0.90 to 1.02 | 0.152 | 0.94 | 0.88 to 0.99 | 0.024 | 0.98 | 0.92 to 1.04 | 0.449 |
| Type of trust staff work for: | |  |  |  |  |  |  |  |  |  |  |  |  |
|  | Acute | 1 |  |  | 1 |  |  | 1 |  |  | 1 |  |  |
|  | Mental health | 1.29 | 1.22 to 1.38 | <0.001 | 1.30 | 1.22 to 1.38 | <0.001 | 1.20 | 1.13 to 1.27 | <0.001 | 1.23 | 1.16 to 1.30 | <0.001 |
| Redeployed outside usual role: | |  |  |  |  |  |  |  |  |  |  |  |  |
|  | No | 1 |  |  | 1 |  |  | 1 |  |  | 1 |  |  |
|  | Yes | 1.10 | 0.99 to 1.21 | 0.065 | 1.10 | 1.00 to 1.21 | 0.060 | 1.13 | 1.04 to 1.24 | 0.005 | 1.15 | 1.05 to 1.26 | 0.002 |
| Felt supported by colleagues: | |  |  | 0.601 |  |  | 0.602 |  |  | 0.997 |  |  | 0.618 |
|  | Extremely | 1 |  |  | 1 |  |  | 1 |  |  | 1 |  |  |
|  | Quite a lot | 1.03 | 0.96 to 1.11 | 0.392 | 1.04 | 0.97 to 1.12 | 0.303 | 1 | 0.93 to 1.06 | 0.875 | 0.99 | 0.93 to 1.06 | 0.747 |
|  | Moderately | 1.05 | 0.95 to 1.15 | 0.353 | 1.05 | 0.96 to 1.15 | 0.311 | 0.99 | 0.91 to 1.08 | 0.808 | 1.05 | 0.96 to 1.14 | 0.309 |
|  | A little | 1.01 | 0.88 to 1.15 | 0.927 | 1.01 | 0.89 to 1.16 | 0.863 | 0.98 | 0.87 to 1.11 | 0.800 | 1.06 | 0.93 to 1.20 | 0.377 |
|  | Not at all | 0.87 | 0.67 to 1.13 | 0.279 | 0.89 | 0.68 to 1.15 | 0.364 | 0.97 | 0.75 to 1.24 | 0.777 | 0.94 | 0.74 to 1.21 | 0.638 |
| Felt supported by manager: | |  |  | 0.120 |  |  | 0.140 |  |  | 0.534 |  |  | 0.259 |
|  | Extremely | 1 |  |  | 1 |  |  | 1 |  |  | 1 |  |  |
|  | Quite a lot | 1.02 | 0.94 to 1.10 | 0.636 | 1.02 | 0.95 to 1.10 | 0.584 | 0.96 | 0.89 to 1.03 | 0.243 | 0.99 | 0.92 to 1.06 | 0.693 |
|  | Moderately | 1.03 | 0.94 to 1.13 | 0.523 | 1.03 | 0.94 to 1.13 | 0.479 | 1.01 | 0.93 to 1.10 | 0.769 | 1.05 | 0.97 to 1.15 | 0.209 |
|  | A little | 0.91 | 0.82 to 1.01 | 0.082 | 0.91 | 0.82 to 1.02 | 0.097 | 0.95 | 0.86 to 1.05 | 0.312 | 0.97 | 0.88 to 1.07 | 0.563 |
|  | Not at all | 0.90 | 0.78 to 1.04 | 0.164 | 0.91 | 0.79 to 1.05 | 0.205 | 1.02 | 0.89 to 1.17 | 0.747 | 1.10 | 0.96 to 1.27 | 0.159 |

OR=Odds ratio. CI=Confidence interval. GHQ12=General health questionnaire.
