# Supplementary material for: Predictors of sickness absence and intention to leave the profession among NHS staff in England during the COVID-19 pandemic: a prospective cohort study
Source: BMJ Open. 2025 Jun 4;15(6):e097483. doi: 10.1136/bmjopen-2024-097483 (PMC12142032; doi:10.1136/bmjopen-2024-097483)
Supplement: Supplementary file 2 [file bmjopen-2024-097483.reviewer_comments.pdf]

PEER REVIEW HISTORY

BMJ Open publishes all reviews undertaken for accepted manuscripts. Reviewers are asked to complete a checklist review form and are provided with free text boxes to elaborate on their assessment. These free text comments are reproduced below.

ARTICLE DETAILS

Title (Provisional)

Predictors of sickness absence and intention to leave the profession amongst NHS staff in England during the COVID-19 pandemic: A prospective cohort study

Authors

Scott, Lauren J; Lamb, Danielle; Penfold, Chris; Redaniel, Maria Theresa; Trompeter, Nora; Moran, Paul; Bhundia, Rupa; Greenberg , N; Raine, Rosalind; Wessely, Simon; MADAN, IRA; Aitken, Peter; Rafferty, Anne Marie; Dorrington, Sarah; Morriss, Richard; Murphy, Dominic; Stevelink, Sharon

---

VERSION 1 - REVIEW

**Reviewer**	**1**
**Name**	**Teklu, Rediet**
**Affiliation**	**University of Gondar, Department of Epidemiology and Biostatistics**
**Date**	**13-Jan-2025**
**COI**	**None**

The study provides valuable insights into the impact of workplace support, demographics, and mental health on employees' intentions to leave the profession and patterns of sick leave during and beyond the COVID-19 pandemic. The comprehensive analysis of demographic disparities and the role of support systems offers a strong foundation for actionable organizational improvements. However, the low response rate and potential response bias should be acknowledged, and efforts to improve representativeness in future research are recommended. Greater exploration of subgroup trends and external factors influencing disparities would enhance the findings. Overall, this work makes a meaningful contribution to understanding workforce dynamics and well-being in healthcare settings.

1. **In the abstract section**

- ✓ The design, setting, and participant details provide necessary context but could benefit from specifying whether the survey was self-reported or conducted by interviewers.
- ✓ Clarifying the follow-up process could be more precise by stating the exact range of follow-up times if available.
- ✓ In the conclusion section of the abstract: The emphasis on fostering social connections is insightful, though providing examples of how the NHS might implement this practically would add value.
- ✓ For keywords: Consider adding terms such as "Workforce retention," "Occupational health," or "Burnout" to capture additional critical aspects of the study.

2. Introduction section

- ❖ Line number 6-8 : The references to media and political debates provide relevance, but specifying recent key reports or data sources could strengthen the argument
- ❖ Line number 15: The statement "Poor staffing levels mean remaining staff have more work to do" is clear but could benefit from additional context or evidence explaining the long-term consequences of such work pressure on service delivery and staff morale.
- ❖ Line number 18: Mentioning the Health and Safety Executive’s report broadens the scope beyond the NHS but may be enhanced by integrating specific NHS-related data for consistency and focus.
- ❖ Line number 26: Highlighting the longitudinal nature of the data collection is helpful, though briefly explaining why the baseline and follow-up intervals (6, 12, and 24 months) were chosen would improve clarity.

- ❖ Line number 32-35: A brief mention of how findings could inform NHS policies or workforce strategies might help emphasize the practical significance of the study.
- ❖ Additional consideration: The potential for data to assess a wider range of aspects of staff wellbeing is insightful. However, it would be beneficial to specify examples of these additional aspects to give a clearer sense of the study's broader impact.

3. **Method section**

Setting and Participants

- ➢ Line number 4:*"Eighteen English NHS Trusts were selected for this study..."*: Consider specifying how the diversity in selection criteria (e.g., urban vs. rural, acute vs. mental health) was operationalized to ensure representativeness.
- ➢ Line number 5-6: *"All Trust staff were eligible..."*: Specify whether there were any specific exclusion criteria for participants.
- ➢ Line number 9: *"Trusts were invited to participate via direct emails..."*: Clarify how the response rate or engagement from invited Trusts was tracked.

Data Collection

- ➢ Line number 13: *"Once recruited, Trusts invited all staff to participate in an online survey."* Consider elaborating on the survey distribution process and how anonymity/confidentiality were maintained.
- ➢ Line number 15: *"The baseline data collection period was April 2020 to January 2021..."*: Ensure the rationale for the baseline and follow-up periods is clear, considering external events (e.g., pandemic phases).
- ➢ Line number 23-24: *"Questionnaires were split into two parts..."*: You might want to explain the rationale for separating short and long questionnaires beyond encouraging participation, particularly if it impacted data completeness.

Outcomes

- Line number 33: *"Outcomes were responses to the following statements..."*: Ensure consistency in wording across outcome descriptions and discuss why dichotomizing responses was appropriate.
- For absenteeism outcomes, clarify how estimation was encouraged when participants may not recall exact durations.

Exposure Variables

- Line number 49: *"Exposure variables of interest were recorded in the baseline questionnaire..."*: Consider providing a rationale for selecting these variables a priori, referencing relevant literature or expert guidance.
- Line number 59: *"Mental health status according to the general health questionnaire-12..."*: It would be helpful to specify how the typographical error impacted data reliability and if any sensitivity analysis was conducted.

Statistical Analysis

- Line number 26: *"Predictors of intention to leave current profession..."*: Ensure the decision to use logistic regression models for intention-to-leave outcomes is justified by explaining why dichotomized outcomes were analyzed instead of ordinal models.
- Line number 33: *"Due to the large number of participants with no sick days..."*: Consider explaining the appropriateness of two-part modeling for this dataset.
- Line number 46: *"For categorical variables with more than two categories..."*: Provide a clear explanation for combining categories with small sample sizes, discussing the potential impact on generalizability.

- Line number 51: *"Analyses are all based on complete case data."* Include a rationale for not using multiple imputation techniques for handling missing data.

Ethical Approval

- Line number 1-3: *"Ethical approval for the NHS CHECK study..."*: Consider highlighting how ethical considerations for data protection and informed consent were addressed in the study.
- Including a mention of data storage, management, and security protocols would enhance transparency about participant privacy.

4. **Result section**
    - Line number 12: The response rate of 15% from the baseline questionnaire is relatively low. This could introduce response bias, as those who responded may systematically differ from those who did not.
    - Line number 13: The exclusion of participants who did not complete the 12-month questionnaire reduced the sample size by over half, which may impact the generalizability of findings.
    - Line number 17: Clinical staff (60%) are well represented, but stratifying further comparisons by sub-roles (doctors, nurses, and other clinical roles) is important for meaningful subgroup analysis.
    - Line number 27-31: The distribution of participants expressing varying levels of intention to leave their profession provides useful insights, though additional context on trends over time or comparisons with pre-pandemic data would strengthen interpretation.

- Line number 35-37: The presentation of generalized mean ratios (GMRs) for leave length comparisons is helpful but could benefit from additional visual representation (e.g., box plots) for clarity
- Additional considerations: The authors should discuss potential biases and limitations, such as self-reporting bias and the low response rate.

**Reviewer**	**2**
**Name**	**Maltezou, Helena C.**
**Affiliation**	**National Public Health Organization, Athens**
**Date**	**01-Feb-2025**
**COI**	**None**

---

1. Methods, page 4: Please provide a definition for “COVID-19 risk group” and “COVID-19 risk group membership”. It is not clear for the reader.

2. Results, page 7: You mention ‘8% of participants (1,169/5,868) stated they strongly agreed that they were actively seeking employment outside their current profession; 12% (710/5,868) agreed, 18% (1,052/5,868) neither agreed or disagreed, 18% (1,067/5,868) disagreed, 43% (2,528/5,868) strongly disagreed and 1% (52/5,868) did not answer this question. 12% of participants (731/5,868) said they thought about leaving their profession every day, 12% (726/5,868) thought about it several times per week, 18% (1,044/5,868) several times per month, 29% (1,694/5,868) several times per year, 28% (1,631/5,868) never thought about it, and 1% (42/5,868) did not answer this question”.

It is suggested that you improve the presentation of your data. You need to present them using a text rather than simply providing numbers and %. Please do not duplicate data already presented in Tables but rather try to present the most interesting and important findings.

3. Did you check the role of COVID-19 vaccination on absenteeism? If not, you need to comment accordingly in limitations section.

4. Discussion: it would be interesting to compare your findings with those reported by others.

5. You may want to use the following article in your reference list:

COVID-19 vaccination significantly reduces morbidity and absenteeism among healthcare personnel: A prospective multicenter study.

Maltezou HC et al. Vaccine. 2021 Nov 26;39(48):7021-7027.

**Reviewer**	**3**
**Name**	**Gohar, Basem**
**Affiliation**	**University of Guelph, Population Medicine**
**Date**	**05-Feb-2025**
**COI**	**None**

Thank you for allowing me to read your paper, NHS Staff: Sickness Absence and Intention to Leave the Profession. I thoroughly enjoyed reading the findings. While many of the results were expected, a couple were interesting.

Overall, this paper is well-written and to the point. I would endorse its publication. However, would the authors be open to expanding the discussion section? Particularly in these areas:

1. The results suggest that nurses had more sick leave than doctors. Considering a job-demands model to interpret the results would be helpful. However, could this association be due to gender? Your results suggest that male participants had lower odds of SA. I do not see the doctor demographics, but is it possible that it is a gender effect? It may be both, but this would be noteworthy in your paper.

2. Recognizing that these are 'COVID era' results, it would be necessary for readers to understand that some of these findings are not unique to this period. With nursing staff representing a large portion of the sample, it would be helpful to advise the readers how the nursing profession is highly vulnerable to sick leaves due to various organizational reasons that intersect with individual characteristics, irrespective of COVID.

3. Age appears to be a robust predictor of adverse outcomes where older workers tend to take fewer sick leaves or even experience poorer mental health, especially during the pandemic (clinical and non-clinical groups, quantitative and qualitative). This begs the question, "Are younger workers (e.g., Gen Z'ers), less resilient? Or is it a preparedness to practice issue?" It would be helpful to embed this in your discussion as a consistent finding.

4. One finding that stood out was related to cultural background, specifically participants of Asian descent. However, this was not addressed in the discussion. There is a meta-analysis from the Lancet that points out that patients who are of Asian ethnicity are at higher risk of COVID (e.g., Size et al., 2020). I am curious if this could be a factor or if it could be possible "psychological safety" fears due to potential discrimination (e.g., Kantamneni, 2020).

5. Unionized vs. Non-Unionized – are all workers unionized? Are there any who are not, and thus, might sick leave be lower for them as perhaps there is job security issue?

Additional comments:

1. The title must include the timeframe, at least indicating that it was during the pandemic.

2. Why was the continuous data categorized (age?)

3. “If a participant had completed 10 or 11 out of the 12 questions, then the most common score across the cohort for the given question was imputed for the remaining one or two questions.” Out of curiosity, how many cases had to be autofilled? Also, what happened to those that only filled, let’s say, 8 or 9? It’s not uncommon to do this, especially with an N this large, but for transparency, does this need to be addressed in the limitation section?

4. This is minor comment, but the tone of the paper is slightly informal; however, I leave this to the editor as it could be catering to an open-access audience.

Thank you and my best.

VERSION 1 - AUTHOR RESPONSE

Reviewers comments	Responses	Actions
**Reviewer 1:**		
The study provides valuable insights into the impact of workplace support, demographics, and mental health on employees' intentions to leave the profession and patterns of sick leave during and beyond the COVID-19 pandemic. The comprehensive analysis of demographic disparities and the role of support systems offers a strong foundation for actionable organizational improvements. However, the low response rate and potential response bias should be acknowledged, and efforts to improve representativeness in future research are recommended.	We thank Reviewer 1 for their kind comments about the value of our research. We agree that, while higher than most other studies of HCW mental health, our response rate was lower than we would have liked, and this may indeed have introduced some bias. We agree that efforts to improve representativeness in future research are needed, and are in discussions with various professional bodies to identify potential avenues for this.	A sentence has been added to the limitations in the discussion highlighting the potential for responder bias.<br><br>“Only 15% of those who were approached participated in the survey, increasing the likelihood of selection bias. However, we believe this response rate is higher than most surveys conducted during the pandemic.”
Greater exploration of subgroup trends and external factors influencing disparities would enhance the findings.	Further work is underway to explore experiences of lower paid staff and those from an ethnic minority background. However, further detailed analyses are out of scope for the current paper.	No action.
Overall, this work makes a meaningful contribution to understanding workforce dynamics and well-being in healthcare settings.	Thank you.	

**Reviewer 2:**		
1. Methods, page 4: Please provide a definition for "COVID-19 risk group" and "COVID-19 risk group membership". It is not clear for the reader.	The UK Government identified various groups at an increased risk of COVID-19 and its consequences during the pandemic. This informed the COVID-19 risk groups asked about in NHS CHECK.	Details added to the exposure variables section in the methods.<br><br>"COVID-19 risk group membership (yes, no; defined by the question "Do you consider yourself as being in an at-risk group (e.g. elderly, existing health conditions or pregnancy) for the COVID-19 (coronavirus)?"
2. Results, page 7: You mention '8% of participants (1,169/5,868) stated they strongly agreed that they were actively seeking employment outside their current profession; 12% (710/5,868) agreed, 18% (1,052/5,868) neither agreed or disagreed, 18% (1,067/5,868) disagreed, 43% (2,528/5,868) strongly disagreed and 1% (52/5,868) did not answer this question. 12% of participants (731/5,868) said they thought about leaving their profession every day, 12% (726/5,868) thought about it several times per week, 18% (1,044/5,868) several times per month, 29% (1,694/5,868) several times per year, 28% (1,631/5,868) never thought about it, and 1% (42/5,868) did not answer this question".<br>It is suggested that you improve the presentation of your data. You need to present them using a text rather than simply providing numbers and %. Please do not duplicate data already presented in Tables but rather try to present the most interesting and important findings.	Thank you for this helpful comment. We agree that these descriptive characteristics are best summarised in a table which we have now added (new Table 2).	We have reduced the detail in the text and have referred readers to the new Table 2.
3. Did you check the role of COVID-19 vaccination on absenteeism? If not, you need to	Unfortunately, we did not enquire about vaccination status. However, once the vaccination was approved it	We have added this as a limitation in the discussion:

comment accordingly in limitations section.	was mandated in NHS workers.	"A final limitation is that we did not collect vaccination status so were unable to assess its impact on sick leave."
4. Discussion: it would be interesting to compare your findings with those reported by others.	We had already done this, however we had missed a heading for the section. This has now been added, along with a couple of additional comparisons to other literature in light of other reviewer comments.	Comparison to other literature section title added.
5. You may want to use the following article in your reference list:<br>COVID-19 vaccination significantly reduces morbidity and absenteeism among healthcare personnel: A prospective multicenter study. Maltezou HC et al. Vaccine. 2021 Nov 26;39(48):7021-7027.	Thank you for this reference.	We have now discussed and referenced this study in the discussion.<br><br>"Interestingly, we found a much lower rate of sickness absence (median 0 days [IQR 0-5] for non-covid related sickness absence and median 0 days [IQR 0 to 0] for Covid-19 related absence) than another cohort study in Greece looking at HCP absence in vaccinated (mean 6.9days, SD 5.7) and unvaccinated (mean 11.9days, SD 8.1) workers over a 5-month period November 2020-April 2021. This may have been due to participants under-reporting sick leave in our study, or perhaps simply higher rates of absence in Greece compared to the UK during this pandemic period."
**Reviewer 3:**		
Overall, this paper is well-written and to the point. I would endorse its publication. However, would the authors be open to expanding the discussion section? Particularly in these areas:	Thank you. Please see responses below.	

1. The results suggest that nurses had more sick leave than doctors. Considering a job-demands model to interpret the results would be helpful. However, could this association be due to gender? Your results suggest that male participants had lower odds of SA. I do not see the doctor demographics, but is it possible that it is a gender effect? It may be both, but this would be noteworthy in your paper.	This is an interesting point. However, all models were adjusted for gender (along with age, ethnicity and session of the 12-month questionnaire completion), so any effect of job role would be over and above the effect of gender.	No action.
2. Recognizing that these are 'COVID era' results, it would be necessary for readers to understand that some of these findings are not unique to this period. With nursing staff representing a large portion of the sample, it would be helpful to advise the readers how the nursing profession is highly vulnerable to sick leaves due to various organizational reasons that intersect with individual characteristics, irrespective of COVID.		The following has been added to the discussion around nursing staff and sickness:<br><br>"In addition, high levels of sickness amongst nursing staff has been widely reported outside of the Covid-19 pandemic."
3. Age appears to be a robust predictor of adverse outcomes where older workers tend to take fewer sick leaves or even experience poorer mental health, especially during the pandemic (clinical and non-clinical groups, quantitative and qualitative). This begs the question, "Are younger workers (e.g., Gen Z'ers), less resilient? Or is it a preparedness to practice issue?" It would be helpful to embed this in your discussion as a consistent finding.		We have expanded our discussion around age, in addition to the existing sentence about health worker survivor bias.<br><br>"Further, it seems likely that younger employees who are unhappy in their work would be more likely to seek alternative employment than older staff who may be contemplating retirement."
4. One finding that stood out was related to cultural background, specifically participants of Asian descent. However, this was not addressed in the discussion. There is a meta-analysis from the Lancet that points out that patients who are of Asian	Participants of all non-white ethnicities were at higher risk of taking COVID-19 sick days and this is already discussed in the 5th paragraph of the discussion.	No action.

ethnicity are at higher risk of COVID (e.g., Size et al., 2020). I am curious if this could be a factor or if it could be possible "psychological safety" fears due to potential discrimination (e.g., Kantamneni, 2020).	The point made about discrimination is important, however, it is beyond the scope of this paper.	
5. Unionized vs. Non-Unionized – are all workers unionized? Are there any who are not, and thus, might sick leave be lower for them as perhaps there is job security issue?	In the UK, not all workers are unionised, and this is not a question we included in our survey, so do not have any data on this. Sick leave is not dependent on union membership, as the same policy applies across all employees.	No action.
Additional comments:<br>1. The title must include the timeframe, at least indicating that it was during the pandemic.		Title amended to "Predictors of sickness absence and intention to leave the profession across NHS staff in England during the COVID-19 pandemic: A prospective cohort study"
2. Why was the continuous data categorized (age?)	Age was not linearly associated with some of the outcomes. For ease of interpretation for the reader, we therefore chose to categorise age rather than adding additional squared terms or similar, which would then not be easily interpretable.	No action.
3. "If a participant had completed 10 or 11 out of the 12 questions, then the most common score across the cohort for the given question was imputed for the remaining one or two questions." Out of curiosity, how many cases had to be autofilled? Also, what happened to those that only filled, let's say, 8 or 9? It's not uncommon to do this, especially with an N this large, but for transparency, does this need to be addressed in the limitation section?	N=296 (2.7%) were imputed according to this rule. For those who completed less than 10 questions, the global score was set to missing.	We have added additional information to the methods about the number of participants for whom this data was imputed.<br><br>"If a participant had completed 10 or 11 out of the 12 questions *(n=296, 2.7%)*, then the most common score across the cohort for the given missing question was imputed for the remaining one or two questions. *If*

		only 1-9 questions (n=44, 0.4%) or zero questions (n=302, 2.8%) were completed, then no imputation was made (and the overall score could not be calculated).”
4. This is minor comment, but the tone of the paper is slightly informal; however, I leave this to the editor as it could be catering to an open-access audience.	This was intended to make it readable to all. However, we are happy for it to be formalised during type setting if the editors require it.	No action.

VERSION 2 - REVIEW

**Reviewer**	**2**
**Name**	**Maltezou, Helena C.**
**Affiliation**	**National Public Health Organization, Athens**
**Date**	**24-Mar-2025**
**COI**	

The authors successfully addressed my previous comments.

**Reviewer**	**3**
**Name**	**Gohar, Basem**
**Affiliation**	**University of Guelph, Population Medicine**
**Date**	**20-Mar-2025**
**COI**	

Thank you for your review and responses. I am content with the updated version, and will leave the "tone" of the paper to the editor. Best wishes to you and the research team.
